# Association between weekend admission and in-hospital mortality for patients with ischemic heart disease upon surgery treatment

**DOI:** 10.3389/fcvm.2024.1435948

**Published:** 2024-10-14

**Authors:** Tianzhao Liu, Chuangpeng Lin, Chenyang Jia, Binbin Wu, Hailong Liu, Yan Liang

**Affiliations:** ^1^ School of Resources and Environment, University of Electronic Science and Technology of China, Chengdu, Sichuan, China; ^2^ Quality Control Department, Peking University Shenzhen Hospital, Shenzhen, Guangdong, China; ^3^ School of Biomedical Sciences, The Chinese University of Hong Kong, Hong Kong, Hong Kong SAR, China; ^4^ Sino-European Center of Biomedicine and Health, Shenzhen, Guangdong, China; ^5^ Wuyi University, Jiangmen, Guangdong, China

**Keywords:** weekend effect, ischemic heart disease, in-hospital mortality, surgery treatment, mediation analysis

## Abstract

**Background:**

The existence and reasons for the weekend effect in patients with ischemic heart disease (IHD) were not yet fully identified. This study aimed to evaluate whether weekend admission was independently associated with in-hospital mortality and the possible mechanisms associated with the IHD patients.

**Methods:**

The study was a retrospective study, including IHD patients from 2015 to 2023. The International Classification of Diseases, tenth revision (ICD-10) codes were used to identify all admissions with a primary diagnosis of IHD. The sample was divided into weekday and weekend groups. We performed a multivariate logistic regression analysis and a mediation analysis to estimate the effect of weekend admission on hospital mortality.

**Results:**

A total of 18,906 IHD patients were included in the study, with an average age of 63.8 ± 12.7. Of these patients, 21.7% (*n* = 4,102) were admitted over the weekend. The in-hospital 30-days mortality rate was significantly higher among the patients admitted at weekends compared with those admitted at weekdays (2.0% vs. 1.1%). Respectively, the 30-day mortality rate of patients admitted on weekends was higher compared to patients admitted on weekdays among patients with surgical treatment (2.34% vs. 1.06%, OR = 1.75; 95% CI: 1.23–2.42) and with emergency admission (3.48% vs. 2.59%, OR = 1.56; 95% CI: 1.05–2.28). Mediation analyses showed that the surgical scheduling had significant mediated effects on the associations of admission time with mortality risk.

**Conclusions:**

IHD patients with a surgical therapy or admitted from emergency department had a significantly higher risk of mortality when admitted on weekends compared to weekdays. These findings have potential implications for resource allocation and redistribution of surgery to weekends in hospitals.

## 1 Introduction

According to the World Health Organization, Ischemic heart disease (IHD) remained a leading cause of mortality worldwide, posing significant challenges to healthcare systems globally (1). Optimal management of patients with IHD, particularly those requiring surgical intervention, was essential for reducing morbidity and mortality rates associated with this disease. However, the timing of admission to the hospital, particularly during weekends, had been associated with variations in patient outcomes across different medical conditions (2–4). This phenomenon, often referred to as the “weekend effect,” had garnered considerable attention in healthcare research. Every patient should have equal access to safe, effective, and high-quality healthcare on any day of the week, and efforts to mitigate the weekend effect are crucial to achieving this goal.

Despite extensive research on the weekend effect across various medical conditions, including acute myocardial infarction (5), stroke (6), and trauma (7), limited studies had specifically examined its impact on patients with IHD undergoing surgical treatment. The weekend admission time was not always associated with an increased risk of hospital mortality (8). The findings regarding the impact of weekend admissions on mortality had been inconsistent in the literature (9, 10). Understanding whether weekend admissions were associated with increased in-hospital mortality rates for patients undergoing surgery for IHD was crucial for improving patient care and optimizing resource allocation (11).

Due to the complexity of medical environmental factors and disease itself, the causes of weekend effect had not been fully elucidated (12). While the underlying mechanisms driving this effect were multifactorial, factors such as reduced staffing levels (13), limited access to specialized services (14), and variations in care processes (15) during weekends had been proposed as potential contributors. Furthermore, existing research showed that the presence of surgery, surgical schedule, and the mode of patient admission (outpatient admissions vs. emergency admissions) played an important role in the occurrence of the weekend effect (16–18). Mohammed et al. (18) reported that patients with weekends admissions have higher risk of death, but this risk was variable among admitted via outpatient department (AO) (OR = 1.32; 95% CI: 1.23–1.41) and admitted via emergency department (AE) (OR = 1.09; 95% CI: 1.05–1.13). It seems to be important to examine whether there was a correlation between admission time, surgical scheduling and in-hospital death and the possible interaction mechanism after controlling for the mode of admission.

This study aimed to investigate the association between weekend admission and in-hospital mortality for patients with IHD undergoing surgical treatment, as well as the possible interaction pathways and mechanisms. Via analyzing a comprehensive dataset of patients undergoing surgical interventions for IHD, we sought to determine whether weekend admissions independently influenced in-hospital mortality rates compared to admissions during weekdays. Identifying and understanding such associations would assist healthcare policies and practices to mitigate the weekend effect and improve outcomes for patients with IHD undergoing surgical treatment.

## 2 Materials and methods

### 2.1 Participants

A retrospective study was performed among IHD patients admitted to Peking University Shenzhen Hospital at the City of Shenzhen, Guangdong Province, P. R. China (PS hospital) from 1 January 2015 to 31 August 2023 and completed discharge procedures (end points: in-hospital death or discharge). The study was approved by the institutional review board of PS hospital. All data were derived from the electronic medical record system (EMR). Data extraction through Structured Query Language (SQL) interface by engineer to ensure the accuracy of the research. Data of patients of IHD, with principal diagnoses codes of I20–25 according to International Classification of Diseases-10 (ICD-10) coding system, were included. Patients with no diagnostic information or hospital-stay duration longer than 30 days were excluded. Due to the retrospective nature of the study, the requirement for informed consent was waived.

### 2.2 Covariates and outcomes

This study defined weekends as non-working days, including Saturdays, Sundays, and statutory holidays in China, totaling approximately 115 days per year. Each day started at 00:00:00 and ended at 23:59:59. Patients were categorized as weekday or weekend admissions based on the admission time recorded in the medical records. Outpatient admissions (AO) referred to patients admitted from the outpatient department to the inpatient department, while emergency admissions (AE) referred to patients transferred from the emergency department to the inpatient department.

The record of the surgery was that of being subjected to the first surgery. The surgical scheduling included elective surgery (ES) or non-elective surgery (NES), surgery on weekday (WDS) or surgery on weekends (WES), and time interval from admission to surgery (TIAS). The primary outcome was the mortality within 30 days of the date of hospital admission.

To account for potential confounding variables, specific covariates—age, sex, mode of admission, level of surgery, hypertension, hyperlipidemia, kidney disease, and history of allergies—were chosen for inclusion in the logistic regression model because prior research had shown that these factors were significant predictors of mortality. To distinguish and analyze age-related cardiovascular risks and their impact on mortality, age was assessed as a categorical variable (<60 years old vs. ≥60 years old). Level of surgery was recorded according to the following categories depending on surgery difficulty: level_1, level_2, level_3, and level_4. The definition and diagnosis of kidney disease are typically based on several key indicators, including a history of chronic kidney disease (CKD), glomerular filtration rate (GFR), serum creatinine levels and whether the patient is undergoing dialysis.

### 2.3 Statistical analysis

Continuous variables were examined for normal distribution and expressed as means ± standard deviation (SD) or median (quartile range), with categorical variables as number (percentage). Quantitative data were compared using student *t-*test or Wilcoxon signed-rank test (for non-normally distributed data). Categorical variables were analyzed by means of the *χ*^2^ test or the Fisher's exact test. Values of overall survival (OS) of the patients were calculated using the Kaplan-Meier method, and the difference among groups was compared using log-rank tests. Multivariate logistic regression model was used to estimate the effect of admission time on the primary outcome. The odds ratios (OR) and the corresponding 95% confidence interval (95% CI) were calculated.

**Figure 1:**
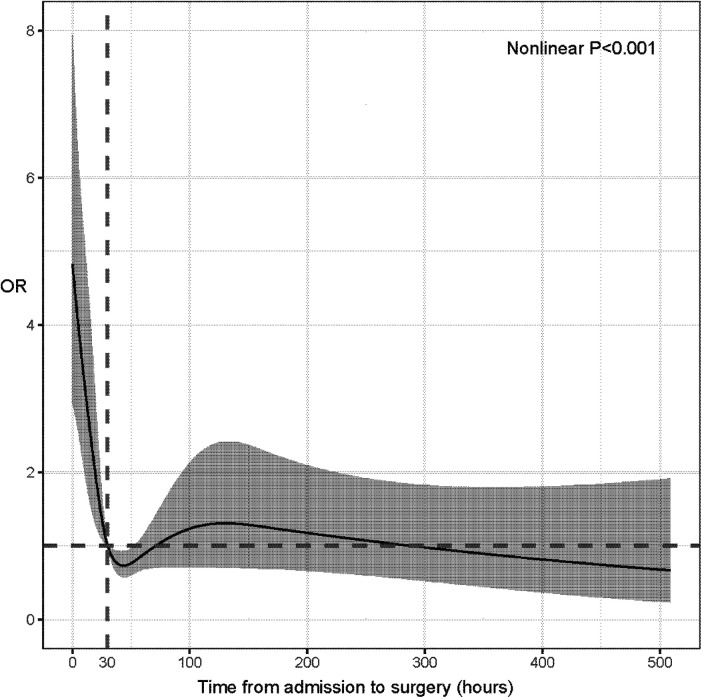
Restricted cubic spline regression for assessing the nonlinear relationship between time interval from admission to surgery and death risk of patients with ischemic heart disease.

Restricted cubic spline regression (19) was conducted to assess the nonlinear relationship between TIAS and the death risk of patients with IHD and determine the optimal cut-off values. The result demonstrated that TIAS had nonlinear relationships with the death risk. Thus, TIAS were converted into binary variables for the following analyses (TIAS < 30 h vs. TIAS ≥ 30 h, Figure 1).

Mediating effects were analyzed by mediation analyses (R package “bruceR”), and bootstrapping technique was used to estimate the confidence interval of the mediating effect (20). The directed acyclic graphs showed the main relationships among exposures, outcomes, and mediators. Two-sided *P* < 0.05 was considered statistically significant. All statistical analyses were performed using R (version 4.3.2; R foundation for statistical computing, Vienna, Austria https://www.R-project.org/).

## 3 Results

### 3.1 Patient characteristics

**Figure 2:**
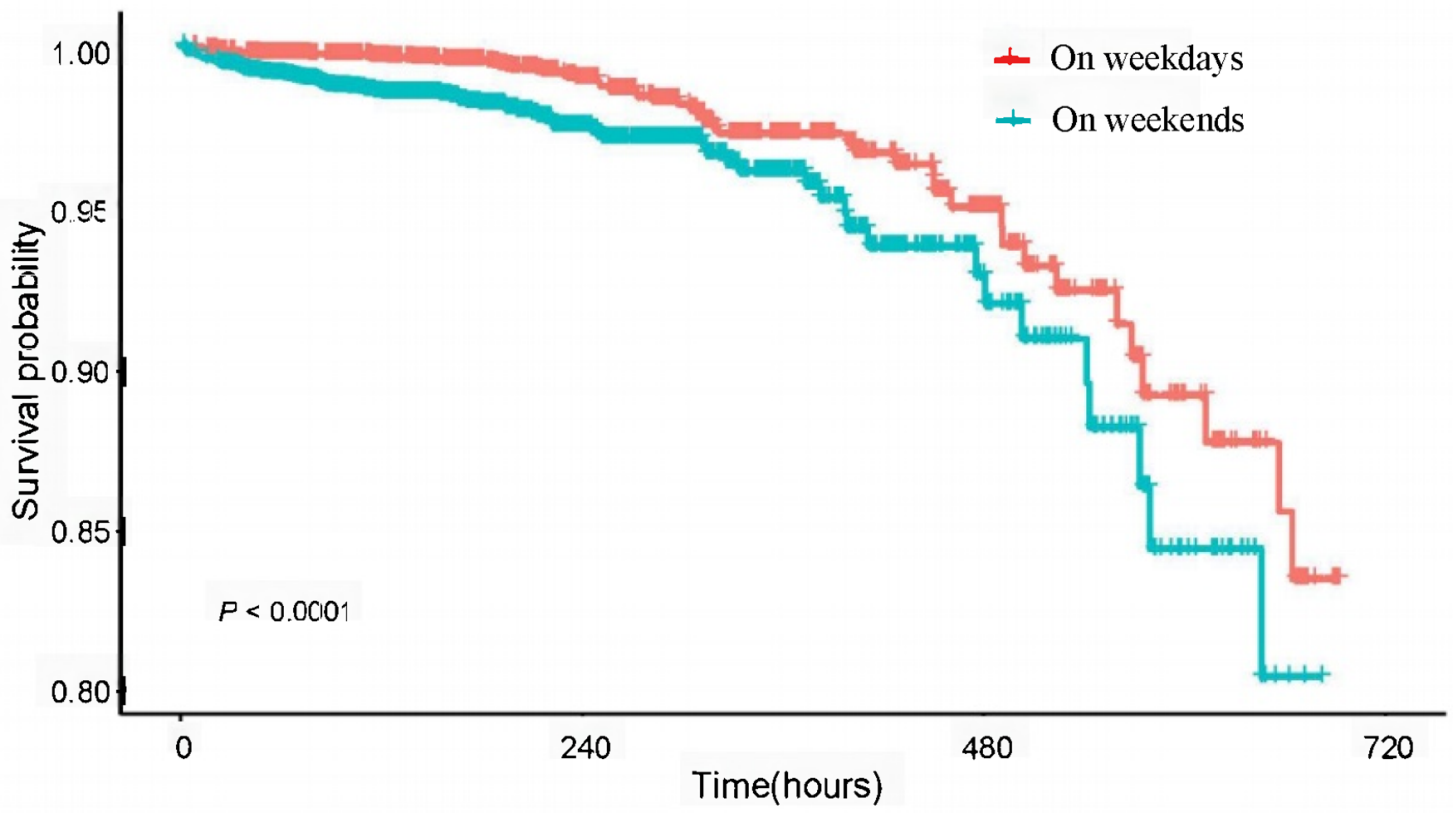
Kaplan-Meier curves of overall survival for admission time in patients with ischemic heart disease.

A total of 18,906 patients were included in this study, with an average age of 63.8 ± 12.7. 239 patients who had a length of stay over 30 days were excluded. Among the total population, 14,804 (78.3%) patients were admitted during working days, while 4102 (21.7%) patients were admitted during weekends. The overall in-hospital 30-days mortality rate was 1.3%, with a 30-day mortality rate of 1.1% for patients admitted working days compared to 2.0% for those admitted weekends *(P* < 0.001). Similarly, the Kaplan-Meier curve showed that the overall survival of patients admitted on weekdays was higher compared with those admitted on weekend (Figure 2). The baseline characteristics of the patients are presented in Tables 1, 2.

**Table 1:** Baseline characteristics of ischemic heart disease patients.

Characteristics	Overall (*N* = 18,906)	Working days admission (*N* = 14,804)	Weekends admission (*N* = 4,102)	*P*
Age:^a^	63.8 (12.7)	63.7 (12.5)	64.1 (13.2)	0.095
Age (Binary):^b^				0.486
<60	6,939 (36.7%)	5,453 (36.8%)	1,486 (36.2%)	
≥60	11,967 (63.3%)	9,351 (63.2%)	2,616 (63.8%)	
Sex:^b^				0.295
Female	6,511 (34.4%)	5,127 (34.6%)	1,384 (33.7%)	
Male	12,395 (65.6%)	9,677 (65.4%)	2,718 (66.3%)	
Marital status:^b^				0.010
Unmarried	209 (1.20%)	148 (1.08%)	61 (1.62%)	
Married	16,845 (96.3%)	13,246 (96.5%)	3,599 (95.6%)	
Other	432 (2.47%)	328 (2.39%)	104 (2.76%)	
Hypertension:^b^				0.037
No	7,356 (38.9%)	5,702 (38.5%)	1,654 (40.3%)	
Yes	11,550 (61.1%)	9,102 (61.5%)	2,448 (59.7%)	
Hyperlipidemia:^b^				0.011
No	12,232 (64.7%)	9,509 (64.2%)	2,723 (66.4%)	
Yes	6,674 (35.3%)	5,295 (35.8%)	1,379 (33.6%)	
Kidney disease:^b^				0.052
No	15,310 (81.0%)	12,032 (81.3%)	3,278 (79.9%)	
Yes	3,596 (19.0%)	2,772 (18.7%)	824 (20.1%)	
Allergy history:^b^				0.016
No	16,860 (89.2%)	13,245 (89.5%)	3,615 (88.1%)	
Yes	2,046 (10.8%)	1,559 (10.5%)	487 (11.9%)	
Mode of admission:^b^				<0.001
Outpatient	12,108 (64.0%)	10,163 (68.7%)	1,945 (47.4%)	
Emergency	6,798 (36.0%)	4,641 (31.3%)	2,157 (52.6%)	
Surgery treatment:^b^				0.006
No	6,159 (32.6%)	4,749 (32.1%)	1,410 (34.4%)	
Yes	12,747 (67.4%)	10,055 (67.9%)	2,692 (65.6%)	
Level of surgery:^b^				<0.001
Level_1	188 (1.47%)	144 (1.43%)	44 (1.63%)	
Level_2	3,642 (28.6%)	2,988 (29.7%)	654 (24.3%)	
Level_3	2,590 (20.3%)	2,095 (20.8%)	495 (18.4%)	
Level_4	6,327 (49.6%)	4,828 (48.0%)	1,499 (55.7%)	
TIAS (Continuous)^a^	52.0 (60.0)	49.8 (59.6)	59.9 (61.1)	<0.001
TIAS (Binary)^b^				<0.001
<30 h	6,365 (49.9%)	5,560 (55.3%)	805 (29.9%)	
≥30 h	6,382 (50.1%)	4,495 (44.7%)	1,887 (70.1%)	
WES:^b^				<0.001
No	10,461 (82.1%)	8,450 (84.0%)	2,011 (74.7%)	
Yes	2,286 (17.9%)	1,605 (16.0%)	681 (25.3%)	
ES:^b^				<0.001
No	1,944 (15.3%)	1,334 (13.3%)	610 (22.7%)	
Yes	10,803 (84.7%)	8,721 (86.7%)	2,082 (77.3%)	

TIAS, time interval from admission to surgery; WES, surgery on weekends, ES, elective surgery.

Data are presented as ^a^mean (standard deviation), ^b^*n* (%) or ^c^median (inter quartile ranges).

**Table 2:** Mortality rates in different groups.

Groups	Sample size	30-day mortality (rate), *n* (%)		*P*
		Working day admission	Weekend admission	
Total population	18,906	165 (1.11%)	83 (2.02%)	<0.001
AO	12,108	45 (0.44%)	8 (0.41%)	0.996
AE	6,798	120 (2.59%)	75 (3.48%)	0.049
Surgical treatment	12,747	107 (1.06%)	63 (2.34%)	<0.001
Non-surgical treatment	6,159	58 (1.22%)	20 (1.42%)	0.656
AO & Surgical treatment	8,126	28 (0.41%)	3 (0.24%)	0.614
AO & Non-surgical treatment	3,982	17 (0.52%)	5 (0.71%)	0.574
AE & Surgical treatment	4,621	79 (2.50%)	60 (4.12%)	<0.001
AE & Non-surgical treatment	2,177	41 (2.78%)	15 (2.14%)	0.459

According to Table 2, among patients with surgical treatment (*n* = 12,747), the 30-day mortality rate of patients admitted on weekends was higher compared to patients admitted on weekdays (2.34% vs. 1.06%, *P* < 0.001). Among patients admitted from the emergency department (*n* = 6,798), the 30-day mortality rate of patients admitted on weekends was higher compared to patients admitted on weekdays (3.48% vs. 2.59%, *P* = 0.049). Among patients with non-surgical treatment or outpatient admission, there was no significant difference in 30-day mortality rates between patients admitted on weekends and on weekdays.

In the population underwent surgery, patients admitted on weekends were more likely to undergo WES (25.3% vs. 16.0%, *P* < 0.001) while patients admitted working days were more likely to undergo ES (86.7% vs. 77.3%, *P* < 0.001). For patients admitted on weekends, a significantly higher proportion of patients experienced TIAS ≥ 30 h compared to the patients admitted working days (70.1% vs. 44.7%, *P* < 0.001).

### 3.2 Mortality risk for patients underwent surgery

Among patients upon surgical treatment, the mortality risk of patients admitted at weekends was higher compared to patients admitted at weekdays (OR = 1.75; 95% CI: 1.23–2.42). Significantly higher mortality risks were observed in patients undergoing WES compared to WDS (OR = 1.96; 95% CI: 1.33–2.71) (Table 3). Conversely, TIAS ≥ 30 h (OR = 0.48; 95% CI: 0.38–0.61) and ES (OR = 0.10; 95% CI: 0.07–0.14) were identified as protective factors against the high mortality risk. Also, patients with a prior medical history of hypertension (OR = 0.49; 95% CI*:* 0.35–0.67) and hyperlipidemia (OR = 0.25; 95% CI*:* 0.15–0.41) exhibited a decreased likelihood of mortality.

**Table 3:** Association between covariates and mortality in IHD patients upon surgery treatment.

Variables	Univariate analysis			Multivariate analysis		
	*β*	OR (95% CI)	*P*	*β*	OR (95% CI)	*P*
Age (Binary):			<0.001			<0.001
<60	–	1.00		–	1.00	
≥60	1.207	3.34 (2.44–4.76)		1.035	2.81 (2.04–4.02)	
Hypertension:			0.005			<0.001
No	–	1.00		–	1.00	
Yes	−0.434	0.65 (0.48–0.88)		−0.720	0.49 (0.35–0.67)	
Hyperlipidemia:			<0.001			<0.001
No	–	1.00		–	1.00	
Yes	−1.723	0.18 (0.1–0.29)		−1.380	0.25 (0.15–0.41)	
Kidney disease:			<0.001			<0.001
No	–	1.00		–	1.00	
Yes	1.804	6.07 (4.48–8.25)		1.513	4.54 (3.30–6.26)	
TIAS:			0.002			<0.001
<30 h	–	1.00		–	1.00	
≥30 h	−0.346	0.71 (0.57–0.88)		−0.729	0.48 (0.38–0.61)	
WES:			<0.001			<0.001
NO	–	1.00		–	1.00	
YES	0.656	1.93 (1.37–2.67)		0.672	1.96 (1.36–2.78)	
ES:			<0.001			<0.001
No	–	1.00		–	1.00	
Yes	−2.143	0.12 (0.09–0.16)		−2.350	0.10 (0.07–0.14)	
Admission time:			<0.001			<0.001
Working day	–	1.00		–	1.00	
Weekend	0.801	2.23 (1.62–3.04)		0.527	1.75 (1.23–2.42)	
Admission time: (WES)^a^			<0.001			<0.001
Working day	–	1.00		–	1.00	
Weekend	0.824	2.35 (1.73–3.69)		0.615	1.85 (1.32–2.58)	
Admission time: (ES)^b^			<0.001			0.005
Working day	–	1.00		–	1.00	
Weekend	0.783	2.09 (1.48–3.05)		0.492	1.63 (1.15–2.30)	
Admission time: (TIAS)^c^			<0.001			<0.001
Working day	–	1.00		–	1.00	
Weekend	0.857	2.55 (1.88–4.15)		0.897	2.45 (1.73–3.44)	

*β*, coefficients; OR, Odds ratio; CI, confidence interval. TIAS: time interval from admission to surgery, WES: surgery on weekends, ES: elective surgery.

^a^ WES was included in multivariate logistic regression analysis.

^b^ ES was included in multivariate logistic regression analysis.

^c^ TIAS was included in multivariate logistic regression analysis.

### 3.3 Effect of admission time on surgical scheduling

The result showed that NES performed on weekends were predominantly carried out on patients admitted on weekends (69.9%), whereas ES on weekends were primarily performed on patients admitted on working days (96.1%). Additionally, patients admitted on weekends undergoing WES exhibited the shortest TIAS (Supplementary Table S1).

The scheduling of ES for patients admitted on weekends primarily occurred on weekdays (74.7%). Additionally, patients admitted on weekends experienced a significantly longer TIAS compared to admitted on working days (Supplementary Tables S2, S3).

The multivariate logistic regression analysis revealed that admitted on working days patients were more inclined to undergo WES (OR = 1.38; 95% CI: 1.25–1.51) and experience longer TIAS (OR = 1.63; 95% CI: 1.51–1.76) (Table 4). Conversely, admitted on weekends patients were less likely to have ES (OR = 0.31; 95% CI*:* 0.28–0.34).

**Table 4:** Association between covariates of basic and surgical scheduling in IHD patients.

Variables	WES			ES			TIAS		
	*β*	OR (95% CI)	*P*	*β*	OR (95% CI)	*P*	*β*	OR (95% CI)	*P*
Age (Binary):			0.034			<0.001			<0.001
<60	–	1.00		–	1.00		–	1.00	
≥60	−0.07	0.93 (0.87–0.99)		0.291	1.34 (1.25–1.43)		0.271	1.31 (1.25–1.38)	
Hypertension:			0.994			<0.001			<0.001
No	–	1.00		–	1.00		–	1.00	
Yes	0.000	1.00 (0.91–1.10)		0.300	1.35 (1.22–1.49)		0.172	1.19 (1.10–1.28)	
Hyperlipidemia:			<0.001			0.002			<0.001
No	–	1.00		–	1.00		–	1.00	
Yes	0.195	1.21 (1.11–1.33)		0.160	1.17 (1.06–1.30)		−0.161	0.85 (0.79–0.92)	
Kidney disease:			<0.001			0.806			<0.001
No	–	1.00		–	1.00		–	1.00	
Yes	0.216	1.24 (1.10–1.40)		−0.017	0.98 (0.86–1.13)		0.367	1.44 (1.31–1.59)	
Admission time:			<0.001			<0.001			<0.001
Working day	–	1.00		–	1.00		–	1.00	
Weekend	0.581	1.79 (1.62–1.98)		−0.645	0.53 (0.47–0.58)		1.082	2.95 (2.69–3.24)	

The data revealed the influence of admission time and surgical scheduling (including WES, ES, and TIAS) on mortality risk in the patients via emergency admission was comparable to that via outpatient admission (Table 5). Among patients with emergency admission, the mortality risk of patients admitted at weekends was higher compared to patients admitted at weekdays (OR = 1.56; 95% CI: 1.05–2.28). However, for outpatient admission, admission time and surgical scheduling posed insignificant effect on mortality risk (*P* > 0.05). In other words, no evidence of a “weekend effect” was observed in the outpatient admission subgroup.

**Table 5:** Association between covariates of basic and mortality in subgroups.

Variables	AO			AE		
	*β*	OR (95% CI)	*P*	*β*	OR (95% CI)	*P*
Age (Binary):			<0.001			<0.001
<60	–	1.00		–	1.00	
≥60	1.522	4.58 (1.94–16.71)		0.968	2.63 (1.86–3.87)	
Hypertension:			0.016			<0.001
No	–	1.00		–	1.00	
Yes	−0.900	0.41 (0.19–0.85)		−0.657	0.52 (0.36–0.75)	
Hyperlipidemia:			0.009			<0.001
No	–	1.00		–	1.00	
Yes	−2.682	0.07 (0.004–0.32)		–1.034	0.36 (0.20–0.59)	
Kidney disease:			<0.001			<0.001
No	–	1.00		–	1.00	
Yes	2.167	8.74 (4.13–19.36)		1.217	3.38 (2.34–4.86)	
WES:			0.068			0.022
No	–	1.00		–	1.00	
Yes	0.785	2.19 (0.89–4.93)		0.484	1.62 (1.06–2.44)	
ES:			<0.001			<0.001
No	–	1.00		–	1.00	
Yes	−2.711	0.07 (0.03–0.16)		−1.674	0.19 (0.12–0.28)	
TIAS (Binary)			0.812			<0.001
<30	–	1.00		–	1.00	
≥30	−0.067	0.94 (0.54–1.66)		−0.605	0.55 (0.41–0.72)	
Admission time:			0.371			<0.001
Working day	–	1.00		–	1.00	
Weekend	−0.534	0.58 (0.39–1.60)		0.442	1.56 (1.05–2.28)	
Admission time: (WES)^a^			0.394			0.083
Working day	–	1.00		–	1.00	
Weekend	−0.527	0.59 (0.14–1.71)		0.338	1.40 (0.95–2.05)	
Admission time: (ES)^b^			0.353			0.019
Working day	–	1.00		–	1.00	
Weekend	−0.581	0.55 (0.13–1.65)		0.449	1.57 (1.08–2.30)	
Admission time: (TIAS)^c^			0.363			0.028
Working day	–	1.00		–	1.00	
Weekend	−0.571	0.57 (0.54–1.66)		0.421	1.52 (1.04–2.21)	

^a^ WES was included in multivariate logistic regression analysis.

^b^ ES was included in multivariate logistic regression analysis.

^c^ TIAS was included in multivariate logistic regression analysis.

The impact of admission time remained consistent in both the emergency admission subgroup and the overall population. However, in the outpatient admission subgroup, it was observed that patients admitted on weekends were less inclined to undergo WES (OR = 0.55; 95% CI: 0.45–0.66) (Supplementary Tables S4–S6).

### 3.4 Mediation analyses

**Figure 3:**
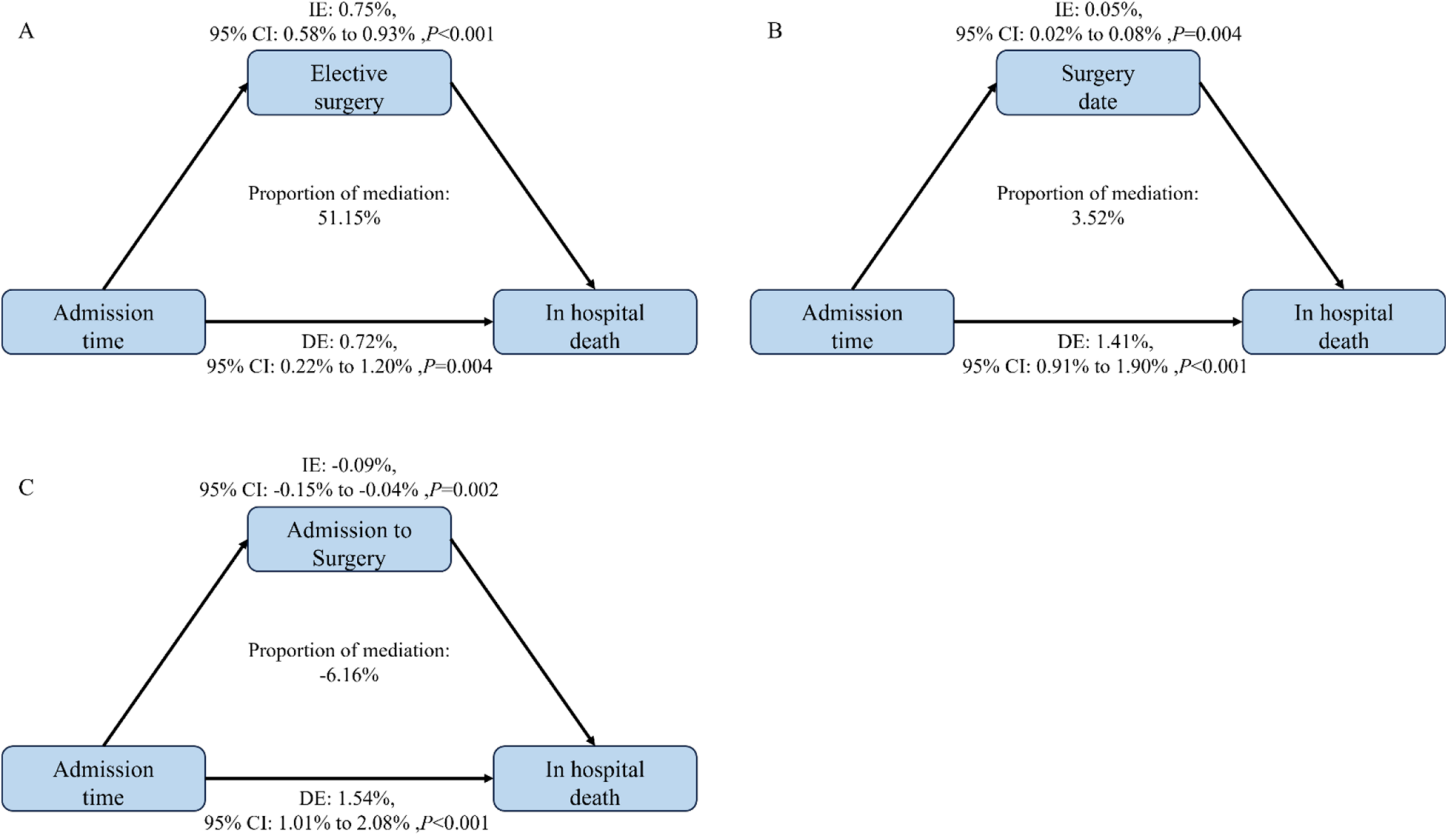
Estimated proportion of the association between admission time and in hospital death by elective surgery **(A)**, date of surgery **(B)** and interval between admission and surgery **(C)** in the patients with ischemic heart disease; proportion of mediation = IE/(IE + DE)* 100%; 95% CI of effect was estimated by bootstrap method (*n* = 1,000).

Mediation analyses showed that ES and WES had significant mediated effects on the associations of admission time with mortality risk, and the proportion of mediation (21) was 51.15% (Figure 3A) and 3.52% (Figure 3B), respectively. Conversely, TIAS had masking effect, and the proportion was −6.16% (Figure 3C). Additionally, in subgroups of emergency admission, ES and WES had significant mediated effects on the associations of admission time with mortality risk, and the proportion of mediation was 18.82% (Supplementary Figure S1A) and 14.24% (Supplementary Figure S1B), respectively. TIAS had masking effect, and the proportion was −6.90% (Supplementary Figure S1C). However, no mediation effects were observed in subgroups of outpatient admission (Supplementary Figures S2).

## 4 Discussion

In this study, we found an overall “weekend effect” in the treatment outcomes of IHD patients admitted to PS Hospital. Further analysis revealed varying degrees of the weekend effect among patients with different characteristics. For instance, surgical patients admitted on weekends had a significantly higher risk of mortality compared to those admitted on weekdays, while non-surgical outpatient patients exhibited no weekend effect. The result demonstrated that we need pay attention to the allocation and utilization of medical resources at different time periods, in order to ensure that patients could receive timely and effective treatment even on weekends.

Surgical patients inherently facing greater risks. In a large multicenter study, WES patients had a higher mortality risk compared to WDS patients (22). Similar findings were reported by McIsaac et al. (23). McIsaac's study identified all residents of Ontario aged 40 years and older who underwent elective, intermediate-risk to high-risk noncardiac surgery between April 1, 2002, and March 31, 2012, with the aim to investigate the association between elective weekend surgery and increased 30-day postoperative mortality. However, the weekend effect was not mentioned in post-cardiac surgery in Sweden since outpatient and emergency admissions were not differentiated (24). In this study, the multivariate logistic regression and the mediation analysis revealed that surgical scheduling influenced patient outcomes, whileas ES and WES served as mediating factors in influencing in-hospital mortality. We found that the patients admitted on weekends were more likely to undergo WES, which was associated with a higher mortality risk. However, this did not imply that WES at all times carried the same mortality risk. The mortality risk for weekend admissions undergoing WES was significantly higher than that for patients admitted on weekdays.

We primarily explored potential reasons of the weekend effect from the perspective of human resources. Typically, each specialty ward during weekends was staffed with only about three attending physicians, responsible for patient observation and various case management throughout the entire ward, leading to potentially reduced quality of consultations and delayed or inaccurate patient assessments. Additionally, the support provided by medical technology departments was crucial. The efficiency of treatments, including medical imaging, ultrasound imaging, laboratory tests and pathology, were reduced during weekends. Severe IHD patients requiring intervention surgeries in the DSA operating room might face delays due to limited anesthesiologists and operating room nurses on duty, leading to treatment delays. Yet, site and equipment appeared not to be reasons causing the weekend effect, as hospital hardware was always available and could be utilized anytime with sufficient personnel. Furthermore, previous studies suggested that less experienced on-duty physicians were associated with higher patient mortality risks (25).

**Figure 4:**
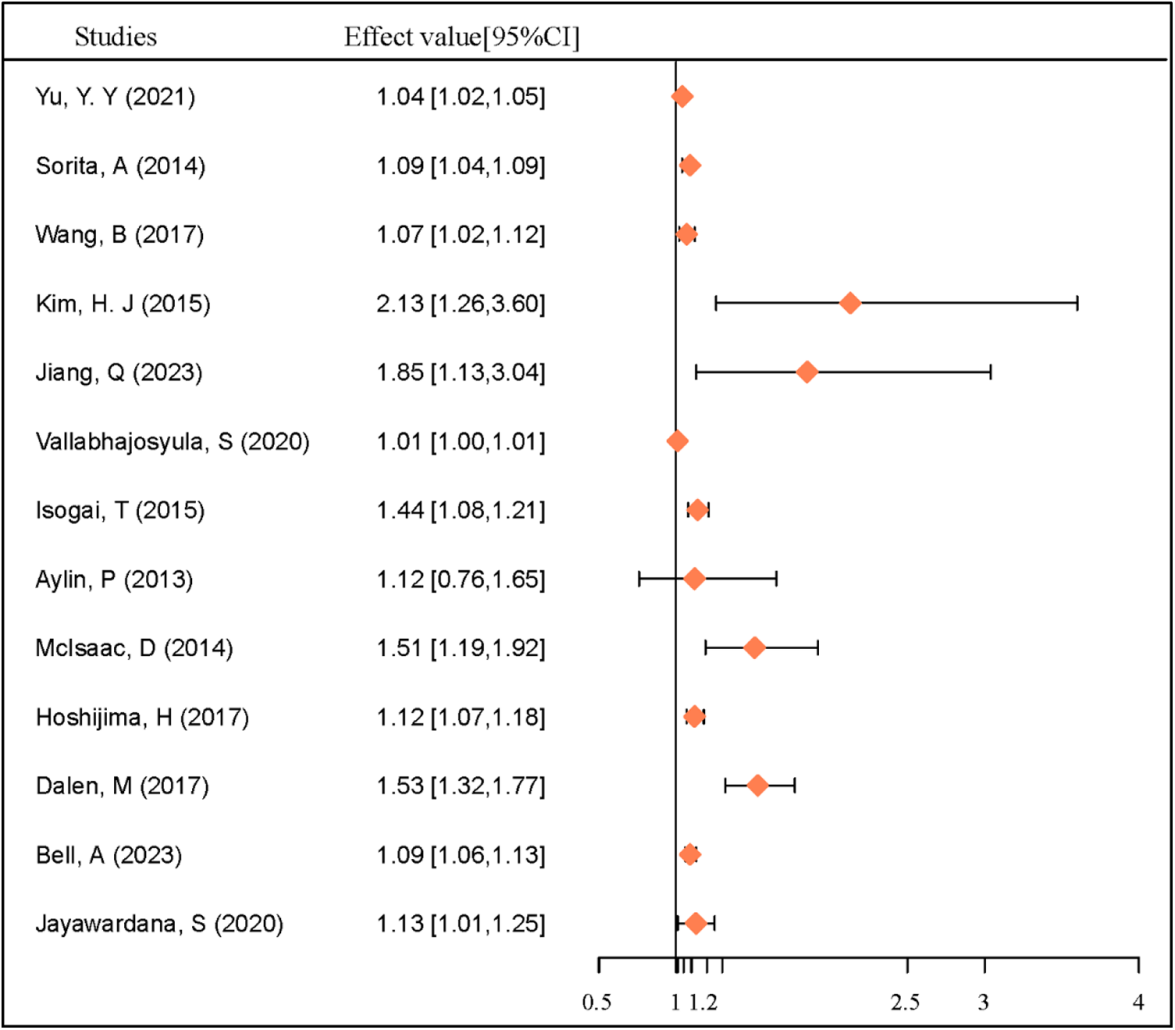
Effect value of weekend effect for heart disease mortality among studies. Effect value: HR (hazard ratio) or OR (odds ratio).

Emergency admissions were found to present more severe symptoms compared to outpatient admissions (5, 26). A meta-analysis including 45 articles and 15,346,544 patients indicated a higher mortality risk for emergency admissions in acute myocardial infarction (AMI) patients (27). Our study indicated that emergency admissions occurring on weekends had a higher mortality rate than those on weekdays. This was consistent with two earlier meta-analyses of AMI (28), and the findings were summarized in Figure 4.

Weekend admissions for emergencies increase mortality risk, due not only to factors mentioned previously but also societal factors. Primary healthcare institutions in Shenzhen, termed Community Healthcare Service (29), had shorter weekend working hours and fewer staff on duty, leading to reduced services compared to weekdays. Consequently, patients tended to have higher trust in large public hospitals in China, leading to a Matthew effect (30), with tiered healthcare systems being less effective on weekends, resulting in increased workloads for emergency departments in large public hospitals.

In this study, there was no significant difference in mortality risk for outpatient patients admitted on weekdays compared with weekends. Disputes existed in similar studies. A study conducted in Australia suggested an increased mortality risk for weekend admissions among emergency patients (31). However, O'Leary et al. found no increased mortality risk for emergency patients admitted on weekends. Conversely, weekend admissions increased mortality risk for outpatient patients. However, O'Leary et al. did not consider cases where patients visited outpatient clinics on weekdays but were admitted on weekends (9).

Outpatient patients generally had more stable conditions and could choose more appropriate admission times. In our subset analysis, the proportion of weekday admissions among outpatient patients was 80.8%, while weekend admissions accounted for 19.2%. Typically, doctors scheduled outpatient admissions on weekdays, but if there were vacant beds on weekends or patients required Monday surgeries, doctors might admit outpatient patients in advance on weekends, leading to no significant difference in mortality rates between outpatient patients admitted on weekends and weekdays.

International literature on the “weekend effect” is inconsistent, and these discrepancies may be due to various factors, including the characteristics of patient cohorts, the organization of healthcare systems, and the availability of medical resources. First, The type and severity of diseases among patients in different studies may vary. The weekend effect may be more pronounced in cases of acute, severe illnesses, as these conditions require timely and complex interventions. If the study cohort mainly consists of patients with chronic or non-acute conditions, the weekend effect might not be as significant. Second, differences in the allocation of medical resources between weekends and weekdays may exist across different countries and regions. Some countries might reduce staffing levels or cut back on key diagnostic and treatment services over the weekend, potentially leading to delays in diagnosis and treatment, which in turn affects patient outcomes. Third, there may also be differences in medical practice guidelines across countries. For example, some countries may have strict time requirements for handling acute conditions, which must be followed even on weekends, while in other places, these guidelines may be less rigorously applied on weekends, leading to treatment delays. The observed differences in the weekend effect in international literature reflect the complexity of global healthcare systems.

This study had several limitations. First, as a retrospective study, although several potential confounding factors were adjusted, some confounding factors possibly still existed, such as the severity of the disease (32), the type of surgery, etc. Studies have shown that coronary artery bypass surgery and percutaneous coronary intervention show different weekend effects (16). Second, this was a single center study, and the location of the study was limited to a general hospital in Shenzhen. In view of the possible heterogeneity of management practices in different hospitals, the generalizability of our findings should be confirmed in a multicentered study. Third, there were some limitations of using electronic medical record data to analyse clinical outcomes. In our observational study we were unable to characterise and analyze the detailed diagnosis and treatment process at an individual patient level to find the root cause of the patient's death.

## 5 Conclusions

In conclusion, this study identified a “weekend effect” in IHD patients at PS Hospital, particularly among those admitted as emergencies. Surgical scheduling played a mediating role in this effect. Patients with both surgical and emergency risk factors had a significantly higher risk of mortality when admitted on weekends compared to weekdays. In light of this situation, hospitals need to establish corresponding multidisciplinary collaborative treatment teams, develop standardized treatment protocols to enhance efficiency, and formulate emergency plans to address unforeseen circumstances. These measures aim to eliminate the disparity in healthcare quality between weekdays and weekends. In fact the ongoing establishment of chest pain centers in China is gradually achieving these goals. Additionally, the uneven distribution of medical resources at the societal level exacerbates the strain on weekend healthcare resources, leading to decreased efficiency and quality of care, which can contribute to various diseases experiencing a weekend effect. As China's tiered healthcare system continues to become more mature, we would continue to monitor changes in hospital weekend effects.

## Data Availability

The original contributions presented in the study are included in the article/Supplementary Material, further inquiries can be directed to the corresponding author.
