# Supplementary material for: Association between weekend admission and in-hospital mortality for patients with ischemic heart disease upon surgery treatment
Source: Front Cardiovasc Med. 2024 Oct 14;11:1435948. doi: 10.3389/fcvm.2024.1435948 (PMC11513328; doi:10.3389/fcvm.2024.1435948)
Supplement: Supplementary file 1 [file Datasheet1.pdf]

Supplementary Material

1 Supplementary Figures and Tables

1.1 Supplementary Figures

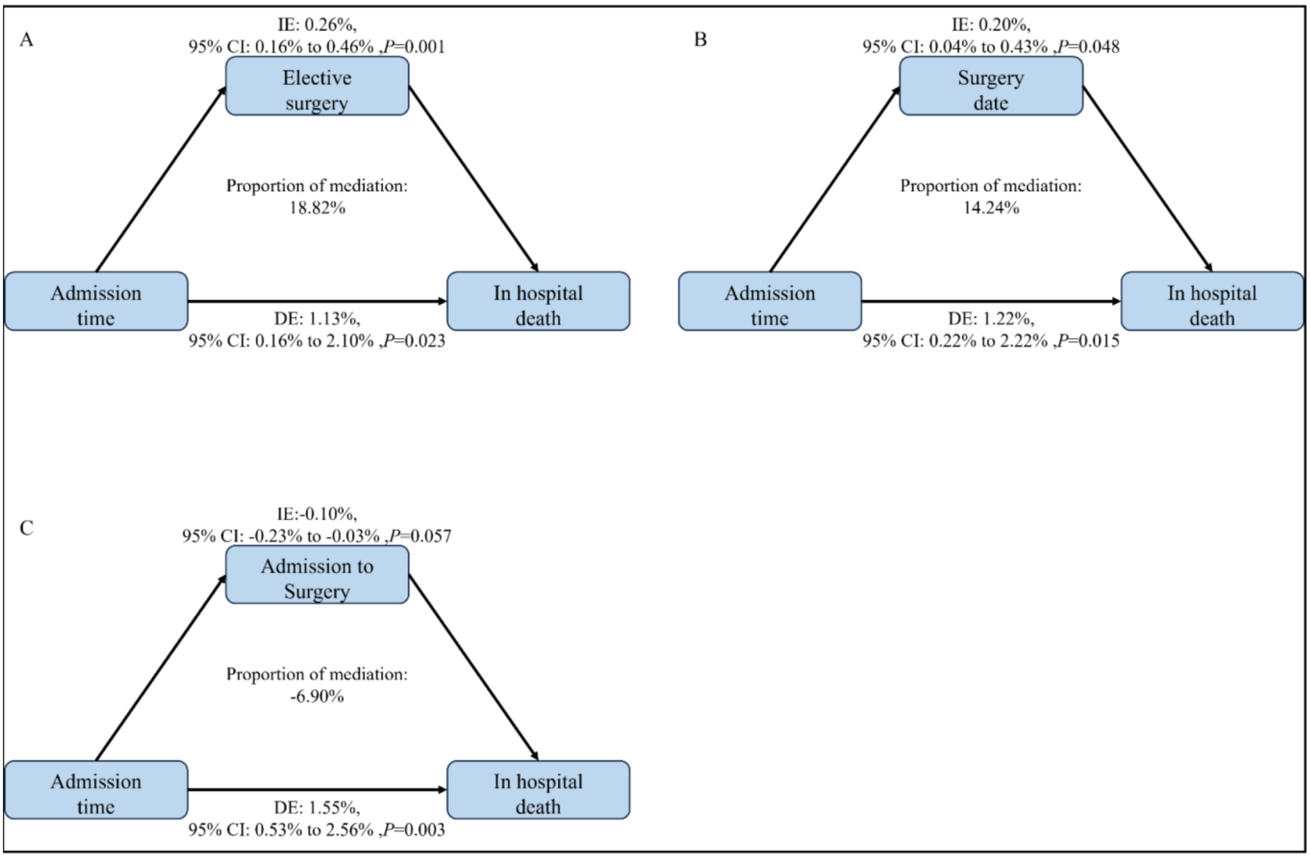

Supplementary Figure S1. Estimated proportion of the association between admission time and in hospital death by elective surgery (A), date of surgery (B) and interval between admission and surgery (C) in the subgroup of emergency admission. IE: indirect effect; DE: direct effect; Proportion of mediation = IE / (IE + DE) * 100%; 95% *CI* of effect was estimated by bootstrap method (*n*=1000).

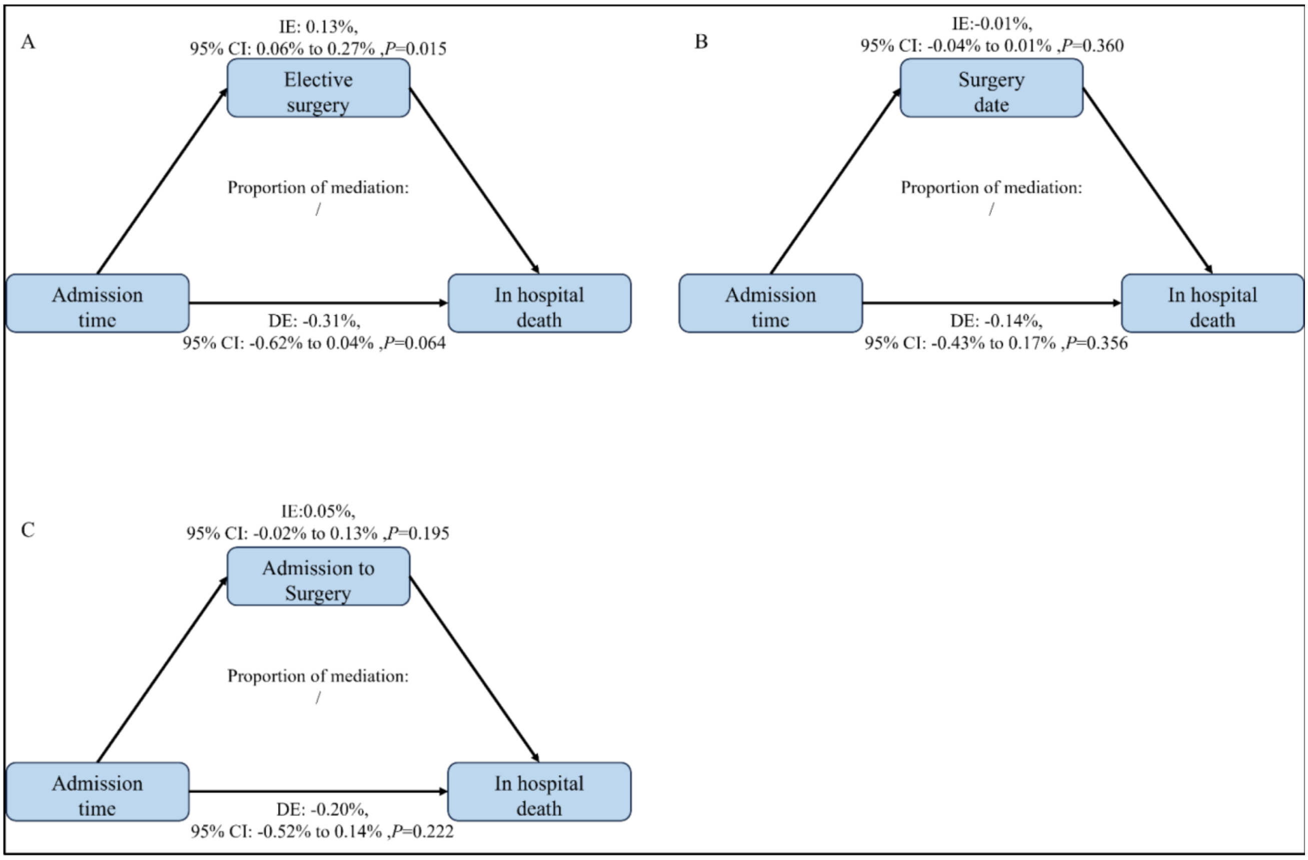

Supplementary Figure S2. Estimated proportion of the association between admission time and in hospital death by by elective surgery (A), date of surgery (B) and interval between admission and surgery (C) in the subgroup of outpatient admission. IE: indirect effect; DE: direct effect; Proportion of mediation = IE / (IE + DE) * 100%; 95% *CI* of effect was estimated by bootstrap method (*n*=1000).

1.2 Supplementary Tables

Supplementary Table S1. The distribution of mode of admission and surgical scheduling in joint grouping of admission time and WES.

**Characteristic**	**Working day admission**		***P***	**Weekend admission**		***P***
	**WDS (*N*=8450)**	**WES (*N*=2011)**		**WDS (*N*=1605)**	**WES (*N*=681)**	
Mode of admission:			<0.001			<0.001
Outpatient	5679 (67.2%)	1210 (75.4%)		1108 (55.1%)	129 (18.9%)	
Emergency	2771 (32.8%)	395 (24.6%)		903 (44.9%)	552 (81.1%)	
ES:			<0.001			<0.001
No	1271 (15.0%)	63 (3.93%)		134 (6.66%)	476 (69.9%)	
Yes	7179 (85.0%)	1542 (96.1%)		1877 (93.3%)	205 (30.1%)	
TIAS (Continuous)	26.8 [16.9;56.6]	34.4 [23.0;51.6]	<0.001	57.1 [45.6;78.8]	3.00 [1.17;14.5]	<0.001
TIAS (Binary)			<0.001			<0.001
<30 hours	4827 (57.1%)	733 (45.7%)		213 (10.6%)	592 (86.9%)	
≥30 hours	3623 (42.9%)	872 (54.3%)		1798 (89.4%)	89 (13.1%)	

The stratification by a combination of admission time and surgery date divided patients into four groups, *P* values are given for $\chi 2$ test for four group comparison. TIAS: time interval from admission to surgery, ES: elective surgery, WES: surgery on weekends, WDS: surgery on weekdays.

Supplementary Table S2. The distribution of mode of admission and surgical scheduling in joint grouping of admission time and ES.

Characteristic	Working day admission		***P***	Weekend admission		***P***
	**NES (*N*=1334)**	**ES (*N*=8271)**		**ES (*N*=610)**	**NES (*N*=2082)**	
Mode of admission:			<0.001			<0.001
Outpatient	203 (15.2%)	6686 (76.7%)		1237 (46.0%)	62 (10.2%)	
Emergency	1131 (84.8%)	2035 (23.3%)		1455 (54.0%)	548 (89.8%)	
WES:			<0.001			<0.001
NO	1271 (95.3%)	7179 (82.3%)		2011 (74.7%)	134 (22.0%)	
YES	63 (4.72%)	1542 (17.7%)		681 (25.3%)	476 (78.0%)	
TIAS (Continuous)	1.85 [1.01;8.83]	30.1 [22.3;68.8]	<0.001	50.0 [22.0;73.9]	1.70 [0.99;9.09]	<0.001
TIAS (Binary)			<0.001			<0.001
<30 hours	1221 (91.5%)	4339 (49.8%)		805 (29.9%)	548 (89.8%)	
≥30 hours	113 (8.47%)	4382 (50.2%)		1887 (70.1%)	62 (10.2%)	

The stratification by a combination of admission time and elective surgery divided patients into four groups, *P* values are given for $\chi 2$ test for four group comparison. TIAS: time interval from admission to surgery, ES: elective surgery, NES: non-elective surgery, WES: surgery on weekends.

Supplementary Table S3. The distribution of mode of admission and surgical scheduling in joint grouping of admission time and TIAS.

Characteristic	Working day admission		***P***	Weekend admission		***P***
	**TIAS< 30 hours (*N*=5560)**	**TIAS ≥ 30 hours (*N*=4495)**		**TIAS < 30 hours (*N*=805)**	**TIAS ≥ 30 hours (*N*=1887)**	
Mode of admission:			<0.001			<0.001
Outpatient	3539 (63.7%)	3350 (74.5%)		178 (22.1%)	1059 (56.1%)	
Emergency	2021 (36.3%)	1145 (25.5%)		627 (77.9%)	828 (43.9%)	
ES:			<0.001			<0.001
No	1221 (22.0%)	113 (2.51%)		548 (68.1%)	62 (3.29%)	
Yes	4339 (78.0%)	4382 (97.5%)		257 (31.9%)	1825 (96.7%)	
WES:			<0.001			<0.001
NO	4827 (86.8%)	3623 (80.6%)		213 (26.5%)	1798 (95.3%)	
YES	733 (13.2%)	872 (19.4%)		592 (73.5%)	89 (4.72%)	

The stratification by a combination of admission time and time interval between admission and surgery divided patients into four groups, *P* values are given for $\chi 2$ test for four group comparison. TIAS: time interval from admission to surgery, WES: surgery on weekends, ES: elective surgery.

Supplementary Table S4. Association between covariates of basic and surgical scheduling in AO subgroups.

**Variables**	**WES**			**ES**			**TIAS**		
	β	**OR (95% CI)**	***P***	***β***	**OR (95% CI)**	***P***	***β***	**OR (95% CI)**	***P***
Age (Binary):			0.216			0.150			<0.001
<60	-	1.00		-	1.00		-	1.00	
≥60	-0.054	0.95 (0.87- 1.03)		0.129	1.14 (0.95-1.36)		0.224	1.25 (1.17-1.34)	
Hypertension:			0.106			0.467			0.071
No	-	1.00		-	1.00		-	1.00	
Yes	0.101	1.11 (0.98- 1.25)		0.093	1.10 (0.85-1.41)		0.087	1.09 (0.99-1.20)	
Hyperlipidemia:			0.003			<0.001			<0.001
No	-	1.00		-	1.00		-	1.00	
Yes	0.182	1.20 (1.06- 1.35)		0.579	1.78 (1.36-2.36)		-0.209	0.81 (0.74-0.89)	
Kidney disease:			0.210			0.309			<0.001
No	-	1.00		-	1.00		-	1.00	
Yes	0.189	1.21 (1.03- 1.42)		-0.170	0.84 (0.61-1.18)		0.560	1.75 (1.53-2.00)	
Admission time			<0.001			<0.001			<0.001
Working day	-	1.00		-	1.00		-	1.00	
Weekend	-0.601	0.55 (0.45-0.66)		-0.547	0.8 (0.44-0.78)		1.848	6.35 (5.39-7.52)	

TIAS: time interval from admission to surgery, ES: elective surgery, WES: surgery on weekends.

Supplementary Table S5. Association between covariates of basic and surgical variables in AE subgroups.

Variables	WES			ES			TIAS		
	β	***OR* (95% *CI*)**	***P***	***β***	***OR* (95% *CI*)**	***P***	***β***	***OR* (95% *CI*)**	***P***
Age (Binary):			0.291			<0.001			<0.001
<60	-	1.00		-	1.00		-	1.00	
≥60	-0.059	0.94 (0.85- 1.05)		0.273	0.31 (1.20-1.43)		0.309	1.36 (1.25-1.49)	
Hypertension:			0.080			<0.001			<0.001
No	-	1.00		-	1.00		-	1.00	
Yes	-0.136	0.87 (0.75- 1.02)		0.313	0.37 (1.21-1.55)		0.268	1.31 (1.16-1.48)	
Hyperlipidemia:			0.002			0.69			0.001
No	-	1.00		-	1.00		-	1.00	
Yes	0.247	1.28 (1.09- 1.50)		-0.026	0.97 (0.86-1.11)		-0.159	0.85 (0.75-0.97)	
Kidney disease:			0.004			0.006			0.005
No	-	1.00		-	1.00		-	1.00	
Yes	0.279	1.32 (1.09- 1.60)		0.230	1.26 (1.07-1.48)		0.223	1.25 (1.07-1.46)	
Admission time:			<0.001			0.286			<0.001
Working day	-	1.00		-	1.00		-	1.00	
Weekend	1.463	4.32 (3.72-5.02)		-0.071	0.93 (0.82-1.06)		0.879	2.51 (2.12-2.74)	

TIAS: time interval from admission to surgery, ES: elective surgery, WES: surgery on weekend

Supplementary Table S6. Baseline characteristics of ischemic heart disease patients upon surgery treatment in subgroups.

Characteristic	Outpatient admission(*N*=8126)			Emergency admission(*N*=4621)		
	Working day admission (*N*=6889)	Weekend admission (*N*=1237)	*P*	Working day admission (*N*=3166)	Weekend admission (*N*=1455)	*P*
Age: [c]	62.0 [54.0;69.0]	63.0 [55.0;70.0]	0.033	61.0 [52.0;71.0]	61.0 [52.0;71.0]	0.659
Age (Binary): [b]			0.043			0.404
< 60	2810 (40.8%)	466 (37.7%)		1406 (44.4%)	666 (45.8%)	
≥ 60	4079 (59.2%)	771 (62.3%)		1760 (55.6%)	789 (54.2%)	
Sex: [b]			0.926			0.282
Female	2204 (32.0%)	398 (32.2%)		872 (27.5%)	378 (26.0%)	
Male	4685 (68.0%)	839 (67.8%)		2294 (72.5%)	1077 (74.0%)	
Marry: [b]			0.306			0.459
Unmarried	62 (0.95%)	15 (1.29%)		61 (2.06%)	35 (2.57%)	
Married	6303 (96.8%)	1116 (96.0%)		2817 (95.1%)	1285 (94.2%)	
Other	145 (2.23%)	32 (2.75%)		85 (2.87%)	44 (3.23%)	
Hypertension: [b]			0.435			0.098
No	2689 (39.0%)	498 (40.3%)		1335 (42.2%)	652 (44.8%)	
Yes	4200 (61.0%)	739 (59.7%)		1831 (57.8%)	803 (55.2%)	
Hyperlipidemia: [b]			0.210			0.582
No	4149 (60.2%)	769 (62.2%)		2037 (64.3%)	949 (65.2%)	
Yes	2740 (39.8%)	468 (37.8%)		1129 (35.7%)	506 (34.8%)	
Kidney disease: [b]			0.156			0.686
No	5891 (85.5%)	1038 (83.9%)		2572 (81.2%)	1190 (81.8%)	
Yes	998 (14.5%)	199 (16.1%)		594 (18.8%)	265 (18.2%)	
Allergy history: [b]			0.045			0.048
No	6249 (90.7%)	1099 (88.8%)		2882 (91.0%)	1297 (89.1%)	
Yes	640 (9.29%)	138 (11.2%)		284 (8.97%)	158 (10.9%)	
TIAS(Continuous) [c]	29.4 [22.3;56.2]	55.2 [44.5;77.9]	<0.001	21.4 [2.87;50.8]	43.0 [2.40;71.2]	<0.001
TIAS (Binary) [b]			<0.001			<0.001
<30 hours	3539 (51.4%)	178 (14.4%)		2021 (63.8%)	627 (43.1%)	
≥30 hours	3350 (48.6%)	1059 (85.6%)		1145 (36.2%)	828 (56.9%)	
WES: [b]			<0.001			<0.001
NO	5679 (82.4%)	1108 (89.6%)		2771 (87.5%)	903 (62.1%)	
YES	1210 (17.6%)	129 (10.4%)		395 (12.5%)	552 (37.9%)	
ES: [b]			<0.001			0.215
No	203 (2.95%)	62 (5.01%)		1131 (35.7%)	548 (37.7%)	
Yes	6686 (97.1%)	1175 (95.0%)		2035 (64.3%)	907 (62.3%)	
Level of surgery: [b]			0.001			0.062
Level_ 1	104 (1.51%)	17 (1.37%)		40 (1.26%)	27 (1.86%)	
Level_2	2332 (33.9%)	352 (28.5%)		656 (20.7%)	302 (20.8%)	
Level_3	1536 (22.3%)	277 (22.4%)		559 (17.7%)	218 (15.0%)	
Level_4	2917 (42.3%)	591 (47.8%)		1911 (60.4%)	908 (62.4%)	

Data are presented as [a] mean (standard deviation), [b] *n* (%) or [c] median (inter quartile ranges). TIAS: time interval from admission to surgery, WES: surgery on weekends, ES: elective surgery.
